# Fine-Root Decomposition and Nutrient Return in Moso Bamboo (*Phyllostachys pubescens* J.Houz.) Plantations in Southeast China

**DOI:** 10.3389/fpls.2022.735359

**Published:** 2022-02-07

**Authors:** Yaowen Xu, Runxia Huang, Benzhi Zhou, Xiaogai Ge

**Affiliations:** ^1^Research Institute of Subtropical Forestry, Chinese Academy of Forestry, Hangzhou, China; ^2^Qianjiangyuan Forest Ecosystem Research Station, National Forestry and Grassland Administration of China, Hangzhou, China

**Keywords:** root diameter, litter, mass loss, decomposition period, release, enrichment

## Abstract

Plant fine-root decomposition is an important pathway for the reentry of nutrients into the soil. Studies have mainly focused on the loss of fine-root mass and the release characteristics of major elements, including, C, N, and P, but there are few reports on trace elements. In this study, *in situ* decomposition experiments were conducted to study the dynamic characteristics of mass loss and residual rates of 10 mineral elements in two diameter classes (<2 mm and 2–5 mm) of moso bamboo in the process of fine-root decomposition. The results of the year-long experiment reported herein showed that: (1) fine roots with diameters of less than 2 mm decomposed faster than those with diameters of 2–5 mm; (2) C, N, P, K, Ca, and Mg were released, whereas Fe, Mn, Zn, and Cu were enriched or changed little; (3) decomposition time and root diameter had significant effects on the remaining percentages of C, N, K, Ca, Mg, Mn, Zn, and Cu, and there were interactions among the elements (*P* < 0.05). The remaining percentages of P and Fe were only affected by decomposition time. This is the first comprehensive report on the variation in 10 elements during the fine-root decomposition of moso bamboo. The study expands our understanding of the release of mineral nutrients during fine-root decomposition, laying a solid theoretical foundation for further research on fine-root decomposition and plant–soil nutrient cycling.

## Introduction

Bamboo is one of the most important non-wood forest products in the world and is an excellent alternative for wood production; hence, it is termed, “the second largest forest in the world” (Yang et al., 2021). In China, moso bamboo (*Phyllostachys pubescens* J.Houz.) forests are among the most important plantations in subtropical areas, with 4.56 million hectares, which accounts for 74% of all bamboo forests in the country (Yang et al., 2021). Compared with most other forest types, moso bamboo shows higher biomass productivity and a shorter rotation period (4–5 years) (Buckingham et al., 2011). Indeed, in addition to producing numerous economically valuable products, due to their great primary productivity, moso bamboo forests produce a large amount of litter as well.

An experiment on broadleaf, coniferous, and bamboo litters showed that litter decomposition not only plays a key role in the material cycle of forest ecosystems but also plays an important role in the energy flow in these ecosystems (Dunmei et al., 2018). Besides aboveground litter, fine roots also account for a large proportion of primary productivity distribution in forest ecosystems and play an important role in nutrient cycling (Fujimaki et al., 2008). Furthermore, although fine roots only account for a small part of the total forest biomass (3–30%), they have a large surface area and high-rate physiological activity and are the main organs for tree water and nutrient absorption (Yuan and Chen, 2010). Concomitantly, fine roots grow and turnover rapidly, thus playing an important and active role in the nutrient cycle of trees. As fine roots release nutrients to the soil throughout the year, they therefore contribute as much as 25–80% to the soil organic carbon pool every year and provide 29–225 kg N/hm^–2^ (Usman et al., 2000).

In most forest ecosystems, 40–90% of newly generated roots will die within 1 year but their total growth can account for 50–75% of the forest primary productivity (Hendrick and Pregitzer, 1992). The amount of organic matter transferred to the soil through fine roots is one to several times that transferred by aboveground litter. Therefore, if the production, death, and decomposition of fine roots are ignored, the turnover of soil organic matter and nutrient elements will be underestimated by 20–80% (Vogt et al., 1986). Thus, in order to further understand the nutrient dynamics of forest ecosystems, we should not ignore the study of fine-root decomposition while studying the decomposition of aboveground litter.

The initial contents of different mineral elements in fine roots are different, as are the roles they play in plants. Therefore, the changes in concentration of these elements in the process of fine-root decomposition are also different; furthermore, the main patterns of change in mineral element contents are release–enrichment–release, enrichment–release, and direct release (Blair, 1988). Release occurs mostly at the early stage of fine-root decomposition, and the duration of enrichment varies with the type of element (He et al., 2019). In many cases, enrichment and release occur alternately, which inevitably presents an irregular fluctuation of the remaining ratio of different elements in the process of fine-root decomposition. To date, studies have mainly focused on the mass loss of fine roots and the release characteristics of carbon (C), nitrogen (N), and phosphorus (P), while studies on trace elements are scarce (Xu et al., 2013; Wang et al., 2014). Although the trace element content is small, these elements are of great significance to plant physiology and ecology; therefore, it is necessary to bolster the study of trace elements in order to comprehensively understand the rules of fine-root decomposition and plant nutrient release to the soil.

The Miaoshanwu Experiment Forests of Qianjiangyuan Forest Ecosystem Research Station are located in Zhejiang Province, China. The typical vegetation in this area is subtropical, natural, broad-leaved secondary forest and artificial forests including, moso bamboo forests, Chinese fir forests (*Cunninghamia lanceolata*), and mass on pine forests (*Pinus massoniana*). The moso bamboo forest was planted in this region in the 1960s and the 1970s, and is an artificial forest typical of south China. In this study, the fine roots of moso bamboo were collected from the Miaoshanwu Nature Reserve. The decomposition rate of fine roots of two different diameters and the dynamic changes in different mineral elements during the process of decomposition were analyzed through a 12-month field experiment. The objectives of this study were as follows: (a) to analyze the decomposition rate of fine roots of moso bamboo of different diameters, and (b) to analyze the remaining percentage and release patterns of different nutrient elements during fine-root decomposition of moso bamboo.

## Materials and Methods

### Study Area

**FIGURE 1:**
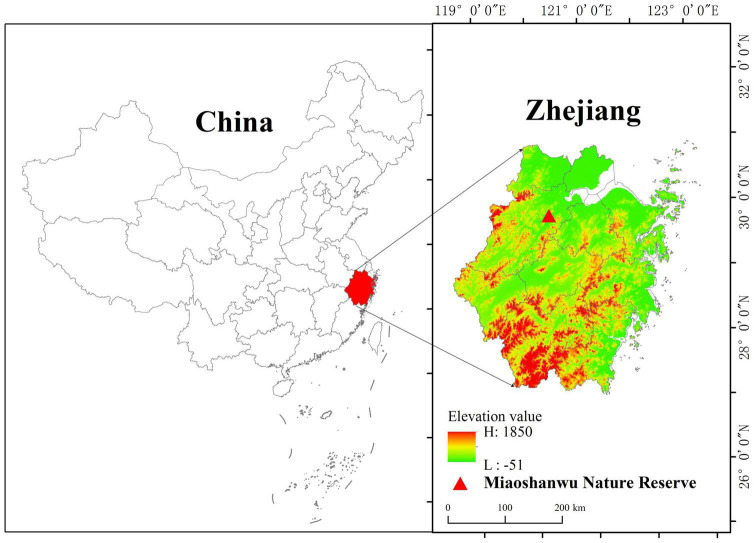
Location of the experimental forest, Miaoshanwu Nature Reserve, Zhejiang province, China.

The study was conducted in the Miaoshanwu Experiment Forests of Qianjiangyuan Forest Ecosystem Research Station, which are located in Fuyang, Zhejiang Province, China (30°03–30°06N; 119°56–120°02E) (Figure 1). The study area belongs to the southeast offshoot of the Tianmushan Mountain Range, a major mountain range in southeast China. The elevation of the area ranges from 70 to 530 m. The region is characterized by an intermediate, subtropical, monsoon, humid climate with a mean annual temperature of 16.1°C. The annual precipitation ranges from 1001.7 to 1964.4 mm, with a mean of 1441.9 mm, which mainly occurs between March and August. The average annual sunshine duration is 1995 h, and the frost-free period is 232 days per year. The soil is a yellowish-brown lateritic soil type with a thickness varying between 30 and 100 cm. The slope varies between 15 and 30°. The physicochemical properties of the soil are listed in Table 1. Moso bamboo forests in this area maintain a structure of 1–5 years of age. Moso bamboo stands have diameters of 6–13 cm at breast height and are 7–14 m high, and stand density varies between 1600 and 2200 individuals/ha.

**TABLE 1:** Physicochemical properties (mean ± standard error; *n* = 3) of the sites where the litter decomposition experiment was conducted.

pH	Organic matter (g/kg)	Bulk density (mg/cm^3^)	Total N (mg/g)	Rapidly available P (mg/kg)	Rapidly available K (mg/kg)
5.5 ± 0.6	23.68 ± 3.05	1.36 ± 0.08	1.68 ± 0.51	2.21 ± 0.32	31.24 ± 2.65

### Collection and Treatment of Fine-Root Samples

A representative moso bamboo forest was selected in the Miaoshanwu Experiment Forests, and a 3-m-wide transect along the slope was set up. Approximately 3 kg of plant roots in the 0–20 cm soil layer was collected by the excavation method. The excavated roots were cut and cleaned, and the dark and inelastic dead roots were removed. Then the samples were packed in a self-sealing bag and transported to the laboratory at low temperature for preservation.

In this study, fine roots were divided into two groups: one group was less than 2 mm in diameter (RDC2), and the other group was 2–5 mm in diameter (RDC5) (Fujimaki et al., 2008). The buried litter bag method was adopted to measure fine root mass and element residual ratio (Fujimaki et al., 2008). The collected fine roots were divided into these two groups, washed, naturally air-dried, and then placed in nylon litter bags [1.5-mm mesh, 30 cm × 30 cm, weighing 10 g (9.23 g after drying)]. Eighty such litter bags were prepared (40 for RDC2 and 40 for RDC5). Two homogeneous quadrats (10 m × 10 m) were randomly selected from the selected sample plot and the soil layer at 10–15-cm depth was excavated in each quadrat. Then, the nylon litter bags were laid in the bottom of the soil layer, and the excavated soil and dead branches and leaves were used to cover them. Subsequently, six nylon litter bags (three for RDC2 and three for RDC5) were retrieved randomly every month for 12 months. The remaining fine-root samples in the recovered nylon litter bags were removed, dried naturally, carefully brushed to remove any attached soil particles, and dried to constant mass at 60°C. The samples were then weighed and subjected to chemical composition analysis.

### Chemical Analysis

After the fine-root were removed from the litter bags, the retrieved fine-root mass was oven-dried at 65°C to constant mass and was then weighed. Thereafter, the oven-dried fine-root samples were ground using a mill and were then sieved through a 0.3-mm mesh screen for measurements of the elemental concentrations. The samples were dissolved in a concentrated acidic mixture of HNO_3_-HClO_4_ (5:1, v/v) which was then heated at 160°C for 5 h. C and N of fine roots were determined using an Elementar CHNS analyzer (Vario EL III, Elementar Analyzer Systeme GmbH, Germany); the concentrations of K, Ca, Mg, Mn, Fe, Cu, Zn, and Na were determined using an inductively coupled plasma (ICP-MS, Elan DRC-e, PE, United States and ICP-OES, Optima 5300 DV, PE, United States) (Yue et al., 2016; Zhang et al., 2019).

### Data Analysis

The fine-root decomposition rate constant k was calculated using the following exponential equation (Olson, 1963):


$$y=M_{\text{t}}/M_{0}=a\,e^{-\mathrm{kt}}$$

where M_0_ is the initial dry weight of fine roots (g), M_t_ is the dry weight of fine roots after a period of time (g), t represents the sampling time (months), and a is a fitting parameter.

The half-life and turnover periods of fine-root decomposition were estimated using the *k* value:


$$t{}_{0.5}=ln0.5/\left(-k\right)$$

$$t{}_{0.95}=ln0.05/\left(-k\right)$$

The remaining percentage of the mineral elements at a certain stage were calculated as:


$$R=M_{\text{t}}C_{\text{t}}/\left(M_{0}C_{0}\right)\times 100\%\left(t=1,2,3,4\ldots \ldots \right)$$

where C_0_ is the fine-root initial element concentration (g/kg), C_*t*_ is the fine-root element concentration after a period of time (g/kg), t represents the sampling time (months), and k is the decomposition constant.

One-way ANOVA was used to examine the differences in initial concentration of fine-root elements. Two-way ANOVA was used to examine the effects of fine-root diameter class, decomposition time, and their interaction on the residual rate in the process of nutrient decomposition. All statistical analyses were performed using SPSS (version 23.0; IBM SPSS Statistics for Windows, IBM Corp., Armonk, NY, United States) with a significance level of 0.05.

## Results

### Pattern of Variation of Fine-Root Mass in Litter Bags

**FIGURE 2:**
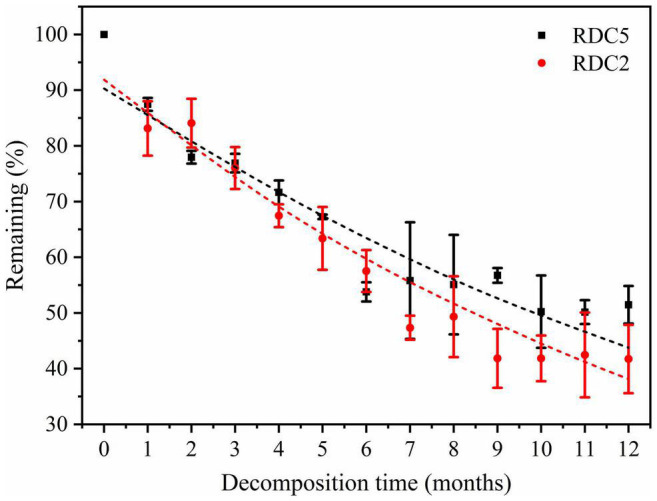
Dry-matter mass loss during fine-root decomposition.

The remaining rates of RDC5 and RDC2 were the same over the first 2 months. After 2 months, the remaining rate gradually diverged until after 12 months when they were 48.53% (RDC2) and 56.26% (RDC5) (Figure 2). As shown in Table 2, the regression analysis showed that the time required for 50% decomposition of dry matter in the RDC5 and RDC2 diameter groups was 11.44 and 9.49 months, respectively. Furthermore, the time required for 95% dry matter decomposition was 49.50 months and 41.10 months, respectively. The decomposition constant rate of fine roots varied among root types (Table 2) with that of RDC2 being higher (*k* = 0.07) than that of RDC5 (*k* = 0.06).

**TABLE 2:** Decomposition parameters and time required for two fine-root diameter groups to decay (t_50_ and t_95_ dry matter loss).

Diameter class	Time of decomposition	Time of decomposition	*R* ^2^	*k*
	50% (months)	95% (months)		
RDC5	11.44	49.50	0.85	0.06
RDC2	9.49	41.10	0.92	0.07

### Characteristics of Initial Content of Nutrient Elements in Fine Roots

As shown in Table 3, in RDC5 fine roots, the initial element concentration from large to small ranked in the following order: C > N > K > P > Mg > Fe > Mn > Zn > Ca > Cu, while in fine roots of the RDC2 group, the initial element concentration from large to small ranked as follows: C > N > K > Fe > P > Mg > Mn > Zn > Cu > Ca. Cu, Fe, Zn, and Mn concentrations in RDC2 fine roots were 5. 42-, 2. 83-, 2. 49-, and 2.12-fold higher than those in the RDC5 group, respectively, and N, K, and Mg concentrations were also higher. In addition, the C/N ratio in RDC2 fine roots was less than that of RDC5 fine roots.

**TABLE 3:** Initial concentration of nutrients in litter from bamboo roots of different diameters.

Root size	C %	N g/kg	P g/kg	K g/kg	Ca g/kg	Mg g/kg
RDC5	46.89 ± 3.12a	6.05 ± 1.09a	2.03 ± 0.32a	5.16 ± 0.52a	0.19 ± 0.08a	1.38 ± 0.77a
RDC2	44.41 ± 1.54a	10.45 ± 0.87b	1.99 ± 0.51a	8.86 ± 1.43b	0.12 ± 0.08a	1.67 ± 0.21a
Root size	Fe g/kg	Mn g/kg	Zn mg/kg	Cu mg/kg	C/N ratio	
RDC5	1.05 ± 0.31a	0.10 ± 0.01a	52.95 ± 8.76a	8.33 ± 3.01a	77.54 ± 9.98a	
RDC2	2.97 ± 0.78b	0.22 ± 0.06b	131.92 ± 10.90b	45.15 ± 7.90b	42.49 ± 3.21b	

*Values followed by different letters on the same column indicate significant differences at P < 0.05.*

### Dynamic Patterns of Different Root Elements in Litter Bags

**FIGURE 3:**
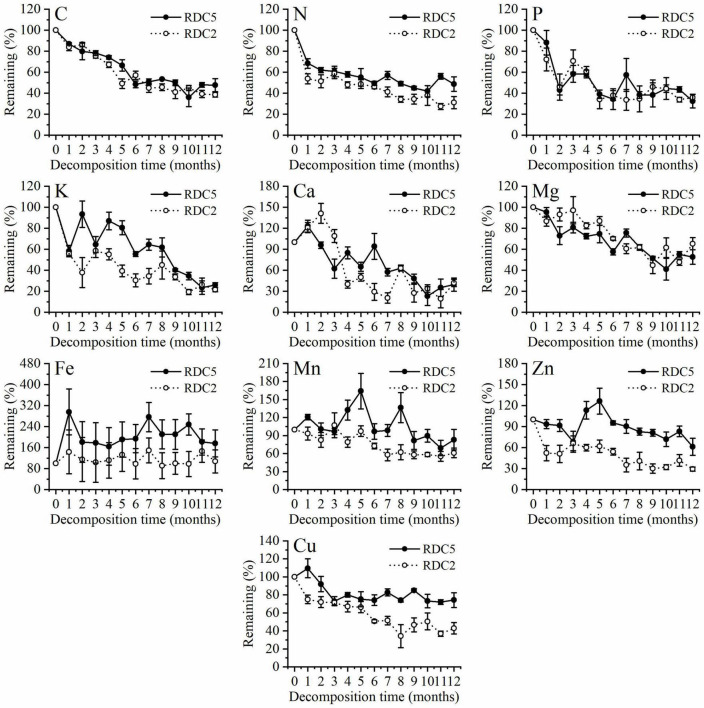
Change trend in element remaining percentage during fine-root decomposition.

Figure 3 shows that in the process of fine-root decomposition, the loss of C was significantly greater in the early stages (1–6 months) than in the late stages (6–12 months). The remaining C of the two diameter classes under study was 86.94% (RDC5) and 83.6% (RDC2) after 1 month, which then gradually decreased (Figure 3). In the seventh month, the remaining C in RDC5 and RDC2 was 50.79 and 44.88%, respectively, after which the loss rate of the remaining C decreased gradually In addition, the change rate of the remaining of C observed for the RDC2 diameter group was slightly lower than that for the RDC5 diameter group.

Figure 3 shows that 1 month after decomposition, the remaining N in the fine roots was 68.33% (RDC5) and 53.8% (RDC2). At 12 months, the remaining N was 48.71% (RDC5) and 38.08% (RDC2). Over 12-months decomposition process the remaining N was always higher in RDC5 than in RDC2 fine roots. In addition, there was a small increase in N remaining in RDC5 at 7 and 10 months.

Figure 3 shows that the remaining P decreased sharply over the first 2 months, with only approximately 45% left in RDC2 and RDC5 at that time, after which the remaining P fluctuated. After 12 months, the remaining P was 32.39% (RDC5) and 35.88% (RDC2).

Similarly, the remaining percentage of K in the two groups of fine-root changed sharply in the early stages, whereas it tended to be stable in the later stages. During fine-root decomposition, the remaining Mg decreased gradually to 52.67% (RDC5) and 65.26% (RDC2) after 12 months (Figure 3).

Figure 3 shows that in the process of fine-root decomposition of the two groups under study here, Ca increased at first and then decreased gradually. After 12 months, the remaining Ca in the two groups of fine roots was approximately 40% and did not differ significantly between groups. The remaining Fe in RDC5 was more than 100% during the experimental period (12 months), indicating enrichment. The remaining Fe in RDC2 fluctuated around 100% during the 12-month experimental period. After 12 months, the remaining Fe of RDC5 and RDC2 was 175.86 and 107.16%, respectively.

Figure 3 shows that the variation in the remaining Mn in the two groups of fine roots differed considerably, with that in RDC5 fluctuating sharply, until 12 months later, when the remaining Mn was 83.13%. The remaining Mn in RDC2 changed more gently and decreased gradually while fluctuating, until after 12 months, when the remaining Mn was 61.09%. In turn, Zn showed a release–enrichment–release dynamic in the two groups.

### Effect of Decomposition Time and Root Diameter on Remaining Percentage

The two-way ANOVA results showed that root diameter and decomposition time had significant effects on C, N, K, Ca, Mg, Mn, Zn, and Cu; furthermore, significant interactive effects for most elements (*P* < 0.05) were detected. Phosphorus and Fe in root material were only affected by time and root diameter, respectively (Table 4).

**TABLE 4:** Two-way ANOVA for decomposition time and root diameter effects on element remaining percentage.

Item	Factor	df	*F*	*P*
C	Root diameter	1	11.344	<0.001
	Decomposition time	11	65.656	<0.001
	Root diameter × Decomposition time	11	3.987	0.001
N	Root diameter	1	73.317	<0.001
	Decomposition time	11	11.724	<0.001
	Root diameter × Decomposition time	11	2.518	0.014
P	Root diameter	1	0.773	0.384
	Decomposition time	11	10.944	<0.001
	Root diameter × Decomposition time	11	1.433	0.19
K	Root diameter	1	91.043	<0.001
	Decomposition time	11	22.112	<0.001
	Root diameter × Decomposition time	11	6.403	<0.001
Ca	Root diameter	1	8.609	0.005
	Decomposition time	11	43.127	<0.001
	Root diameter × Decomposition time	11	11.591	<0.001
Mg	Root diameter	1	10.101	0.003
	Decomposition time	11	25.163	<0.001
	Root diameter × Decomposition time	11	4.247	<0.001
Fe	Root diameter	1	30.4	<0.001
	Decomposition time	11	0.866	0.578
	Root diameter × Decomposition time	11	0.449	0.925
Mn	Root diameter	1	69.438	<0.001
	Decomposition time	11	8.885	<0.001
	Root diameter × Decomposition time	11	3.112	0.003
Zn	Root diameter	1	227.802	<0.001
	Decomposition time	11	8.182	<0.001
	Root diameter × Decomposition time	11	2.512	0.014
Cu	Root diameter	1	175.904	<0.001
	Decomposition time	11	11.85	<0.001
	Root diameter × Decomposition time	11	3.541	0.001

## Discussion

### Mass Loss During Fine-Root Decomposition

The results showed that the decomposition of fine roots of moso bamboo was not constant. Fine-root decomposition is the process of material exchange with the surrounding environment. Fine-root material re-enters the soil under the action of microorganisms (Burton et al., 2000). Some studies suggest that plant decomposition is controlled by the vegetative strategies of fungal groups, which in turn may be controlled by r-type strategists (such as Mucorales) sensitive to fresh organic matter and nutrients in the early stages of decomposition. Then, when the soluble organic matter is exhausted, k-type strategists (such as Eurotiales and Agaricales) decompose refractory organic matter, such as cellulose and lignin, thus controlling the process at this stage (Venkatesagowda, 2019). Therefore, the differences in nutrient strategies and microbial ecological niches during decomposition may be the ultimate cause for the different decomposition rates observed. A previous study showed that fine-root decomposition way inversely correlated to the lignin:N ratio, with fine and small roots showing higher lignin:N ratios (Fujimaki et al., 2008); this may explain why RDC2 had a higher constant decomposition rate. In addition, there are significant differences in N concentration in moso Bamboo roots of different growth stages (Li et al., 1998), which will affect the decomposition of roots. Therefore, in future work, we will study the effects of different growth stages on root decomposition in moso bamboo.

The relationship between decomposition of fine roots and climatic conditions was found to be weak, even though the decomposition of bamboo is related to seasonal variation in precipitation (Fujimaki et al., 2008). Differences in moisture conditions also do not explain faster litter decomposition in a temperate forest, thus differences were speculated to be species-specific and dependent on the initial root chemistry rather than local climate (Fujimaki et al., 2008).

### Release of Nutrient Elements in the Litter Bags

This study showed that the decomposition of RDC2 fine roots was faster than that of the RDC5 group. In general, the thinner the roots, the greater the ratio of surface area to volume, and the larger the surface contact with the surroundings, which is conducive to the invasion and decomposition of microorganisms. Many studies have found that N content decreases and C/N increases with root diameter and substrates with higher C/N denote higher concentrations of structural substances; concomitantly, N is less available and therefore more difficult to decompose (Pregitzer et al., 2002; Yang et al., 2004). Silver and Miya (2001) collected global root data and found that broad-leaved species tend to decompose faster than coniferous species because broad-leaved species have higher N concentrations and lower C/N ratios.

It was found in this study that the remaining of C and N both showed a slight upward fluctuation in the process of gradual decline. C is the main constituent of organic matter, and its release usually shows a linear change with the decrease in litter mass. Furthermore, C showed a direct release mode in a previous study (Moore et al., 2006). Another, study found that N is enriched in the process of root decomposition (Chen et al., 2001). Biological degradation of litter is mainly performed by microorganisms such as fungi and bacteria, which have lower C/N values than most types of litter. Therefore, C and N are only net released in the form of minerals when the C/N values of plant residues reach critical levels (Manzoni et al., 2008). Thus, in this study, the remaining C and N both showed slight upward fluctuation in the process of gradual decline may be trying to sustain the C/N values of plant residues.

As for P, our results showed a release–enrichment–release pattern during fine-root decomposition, as well as a P release pattern during fine-root decomposition. However, evidence has been reported suggesting that P is released during grass root decomposition in a steppe, whereas it tended to be immobilized in forests (Gargaglione et al., 2018). An explanation of this discrepancy may involve the N/P ratio. A previous study reported that litter with N/P > 22 tends to be immobilized during decomposition but litter with a lower N/P ratio may tend to show P immobilization or mineralization (Güsewell and Freeman, 2005). Meanwhile, the concentration of K was fluctuating, and several studies that have shown that K is involved in a typical direct release mode (Edmonds and Tuttle, 2010; Gargaglione et al., 2018). A possible reason is that K is mainly present in the solution of plant cells in the form of ions that are released directly mainly through leaching rather than in the form of structural substances (Osono and Takeda, 2004). The experimental area was located in the subtropical zone with frequent precipitation, and the change in soil water content may be the reason for this phenomenon.

The remaining Ca and Mg in the fine roots of moso bamboo gradually decreased during the alternating process of enrichment and release. Ca and Mg mainly exist in plants in the form of chelates, and at the early stages of decomposition, the chelate macromolecules gradually disintegrate and Ca and Mg are freed. With the increase in temperature and rainfall, Ca and Mg were released from the litter (Whitford et al., 1995). Therefore, temperature and precipitation will have a strong impact on the release of Ca and Mg, which may be the reason why the remaining Ca and Mg gradually decreased in the alternating periods of enrichment and release.

This study showed that the remaining metal elements Mn, Mg, and Fe exceeded 50% after 12 months, and these elements are more difficult to release than non-metallic elements. The same phenomenon was found in some leaf decomposition experiments, suggesting that as organic matter components, Mn, Mg, and Fe are difficult for microorganisms to decompose and use which may be the cause of their enrichment in the soil (Berg et al., 2010). To date, the mechanism of the increase in the remainder of some elements during the decomposition of plant residues remains unclear. Other previous studies focused on heavy metal elements in the process of decomposition of plant residues and observed an absolute increase (Edmonds and Tuttle, 2010; Nikolaidou et al., 2010). Some of these reports suggested that this kind of element enrichment during the decomposition process may be controlled by biological and chemical factors, namely, heavy metal elements may become strongly fixed by forming highly stable chelates as part of the chemical formation of humic acids (Rustad and Cronan, 1988).

### Reflections and Prospects of Fine-Root Decomposition

**FIGURE 4:**
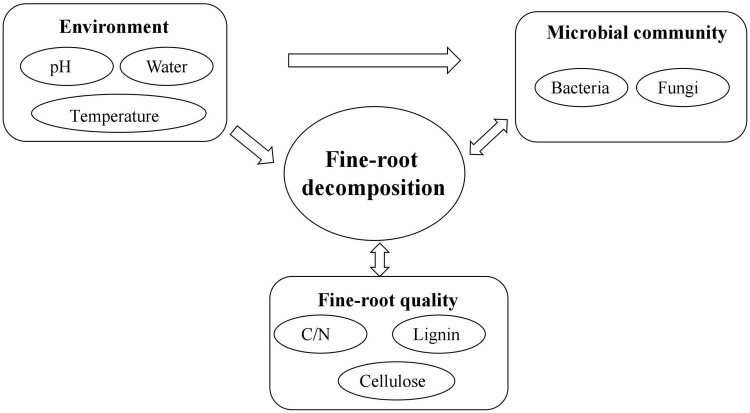
Factors controlling fine-root decomposition and their interactions.

Fine-root decomposition is a complex physical, chemical, and biological process (Figure 4). The factors affecting the decomposition process, the resulting plant elements, and nutrient release include the environment, litter quality and quantity, the microbial community, and the complex interrelationships among these factors (Smith et al., 2014). The extent of their influence is usually ordered as follows: environment > litter quality > microbial community (Aerts, 2006). Previous studies assumed that the environment was the key factor in determining decomposition, while litter quality only played a role when the environmental influence was not evident. In this scheme, the microbial community was thought not to affect the decomposition process directly but to be determined by the environment and litter quality, depending on which, it then exerted an effect on decomposition. However, an increasing number of studies have found that on a very small scale, the microbial community is the key factor directly affecting decomposition (Bradford et al., 2016). Currently, the importance and role of each factor in different ecosystems and at different stages of decomposition are assumed to vary, although the mechanisms are not clear (Gracia-Palacios et al., 2013). Therefore, it is important to pay attention to the study of fine-root decomposition processes at a small regional scale, and to determine the causes and threshold levels of changes in dominant factors of decomposition in future fine root research.

## Conclusion

This is the first extensive report on the changes in the concentrations of 10 mineral elements during fine-root decomposition in a moso bamboo forest. Our experiments showed that initial element concentration and decomposition of fine roots with different diameters are different with finer roots (diameter <2 mm) decomposing faster. In general, C, N, P, K, Ca, and Mg were released, whereas Fe, Mn, Zn, and Cu were either enriched or unchanged to any significant extent. In addition, the release and enrichment of elements during the decomposition process were not linear but rather fluctuated. Our findings thus lay a solid theoretical foundation for further research on the mechanism of plant–soil nutrient cycling and the factors governing the process of fine-root decomposition.

## Data Availability Statement

The raw data supporting the conclusions of this article will be made available by the authors, without undue reservation.

## Author Contributions

BZ contributed to the conception of the study. YX contributed significantly to analysis and manuscript preparation, performed the data analyses, and wrote the manuscript. XG, RH, and BZ helped to perform the analysis with constructive discussions. All authors contributed to the article and approved the submitted version.

## Conflict of Interest

The authors declare that the research was conducted in the absence of any commercial or financial relationships that could be construed as a potential conflict of interest.

## Publisher’s Note

All claims expressed in this article are solely those of the authors and do not necessarily represent those of their affiliated organizations, or those of the publisher, the editors and the reviewers. Any product that may be evaluated in this article, or claim that may be made by its manufacturer, is not guaranteed or endorsed by the publisher.

## Abbreviations

C: carbon
N: nitrogen
P: phosphorus
K: potassium
Ca: calcium
Mg: magnesium
Fe: iron
Zn: zinc
Cu: copper
Mn: manganese.
